# Genistein Affects Histone Modifications on Dickkopf-Related Protein 1 (DKK1) Gene in SW480 Human Colon Cancer Cell Line

**DOI:** 10.1371/journal.pone.0040955

**Published:** 2012-07-18

**Authors:** Huan Wang, Qian Li, Hong Chen

**Affiliations:** Department of Food Science and Human Nutrition, University of Illinois at Urbana-Champaign, Urbana, Illinois, United States of America; UCSF/VA Medical Center, United States of America

## Abstract

Genistein (GEN) is a plant-derived isoflavone and can block uncontrolled cell growth in colon cancer by inhibiting the WNT signaling pathway. This study aimed to test the hypothesis that the enhanced gene expression of the WNT signaling pathway antagonist, DKK1 by genistein treatment is associated with epigenetic modifications of the gene in colon cancer cells. Genistein treatment induced a concentration-dependent G2 phase arrest in the human colon cancer cell line SW480 and reduced cell proliferation. Results from several other human colon cancer cell lines confirmed the growth inhibitory effects of genistein. Overexpression of DKK1 confirmed its involvement in growth inhibition. Knockdown of DKK1 expression by siRNA slightly induced cell growth. DKK1 gene expression was increased by genistein in SW480 and HCT15 cells. DNA methylation at the DKK1 promoter was not affected by genistein treatment in all the cell lines tested. On the other hand, genistein induced histone H3 acetylation of the DKK1 promoter region in SW480 and HCT15 cells. This indicates that increased histone acetylation is associated with the genistein-induced DKK1 expression. The association between histone acetylation and DKK1 gene expression is confirmed by the histone deacetylase inhibitor trichostatin A (TSA) treatment. In conclusion, genistein treatment decreases cell growth and proliferation in colon cancer cell lines. The effect is associated with the increased DKK1 expression through the induction of histone acetylation at the DKK1 promoter region.

## Introduction

Soy contains various bioactive components, which have received much attention in their potential ability to reduce cancer risk [1], [2]. Epidemiological studies showed that consuming higher levels of dietary soy products contributes to the lower incidence of colorectal cancer in Asian countries [3], [4], [5]. Specifically, genistein (4, 5, 7-trihydroxy-isoflavone), a natural isoflavone abundant in soy, has been shown to reduce colorectal cancer risk [6], [7]. These studies provide strong evidence for the need to further investigate the mechanisms behind genistein’s anticancer potential.

In cell culture studies, genistein has been reported to alter cell physiology in several colon cancer cell lines. A recent study performed by Fan et al. identified that colon cancer cells had changed morphology, including chromatin condensation and nuclear fragmentation after genistein treatment [8]. In addition, higher concentrations of genistein of >10 µmol/L significantly induced inhibition of cell proliferation and DNA fragmentation in a human colon cell line [9]. Moreover, in colon cancer cell lines Caco-2 and SW620, cell death was induced by soybean extract treatment [10]. Therefore, genistein is becoming a promising compound in colon cancer prevention and treatment. In the present study, we have further explored the antitumor properties of genistein by testing cell cycle progression and cell proliferation in several colorectal cancer cells in response to treatment with increasing concentrations of genistein.

Various pathways and mechanisms have been proposed to be responsible for genistein’s ability to reduce cancer risk. IGF-IR signaling, the AKT pathway and cell growth regulation are associated with the antitumor effects of genistein [9], [11]. Additionally, genistein also possesses antioxidant properties by mimicking estrogen via estrogen receptor-mediated phosphorylation of ERK1/2 and activation of the NFκB signaling pathway [12]. Further reports from recent studies in animals indicated that genistein inhibited hormone-dependent or -independent cancer cells by regulating interactions between vitamin D and estrogen receptor [13], [14], [15]. Genistein regulates gene transcription in various cancer cell lines by epigenetic regulations, e.g. DNA methylation and histone modifications [16], [17], [18]. Genistein alters the DNA methylation of various genes in rat and mouse models [19], [20]. However, the mechanisms behind genistein’s role in cell proliferation or apoptosis during carcinogenesis remains poorly understood.

The Wingless-int (WNT) signaling pathway comprises a large number of growth factors that are involved in organogenesis, proliferation, regeneration, cell fate determination and cell-cell adhesion [21], [22]. WNT proteins bind to Frizzled receptors (FRZ) and low-density lipoprotein receptor-related protein (LRP) co-receptors, causing cytosolic β-catenin stabilization and accumulation. Accordingly, nuclear β-catenin increases and complexes with TCF/LEF transcription factors, leading to the increased transcription of target genes, including cyclin D1 [23]. Aberrant WNT signaling is one of the contributors for the transition from normal colonic epithelium to malignant tumor cells [24], [25]. Genistein was recently reported to suppress WNT signaling in colon cancer cell lines [26], [27], which provides one potential mechanism for genistein’s anticancer capacity.

One of the key regulators of the WNT signaling pathway is its antagonist Dickkopf 1 (DKK1). DKK1 prevents β-catenin-mediated signal transduction by binding to LRP and Kremen proteins, promoting cells differentiation and apoptosis [28], [29], [30], [31], [32]. The silencing or loss of DKK1 has been documented in various diseases. In colorectal cancer silenced *DKK1* expression is tightly associated with microsatellite instability of tumor subtypes [33]. It is reported that the tumor suppressing capacity of DKK1 is repressed by the hypermethylation of its promoter in the advanced stages of colorectal cancer [34]. In human renal carcinoma cells, chemically inducing histone acetylation at the DKK1 promoter resulted in the re-activation of DKK1 expression, demonstrating that in addition to DNA methylation, histone tail modifications might also contribute to the transcriptional control of critical genes related to cancer development [35].

In the present study, we investigated the anticancer properties of genistein by identifying its effects on cancer cell physiology and examining the mechanistic basis of *DKK1* activation. In particular, this study is among the first to link genistein-mediated epigenetic modifications of *DKK1* to the anticancer properties of genistein in colorectal cancer.

## Results

### Genistein Treatment Selectively Induced DKK1 Gene Expression but did not Alter DKK1 Promoter Methylation Patterns

**Figure 1:**
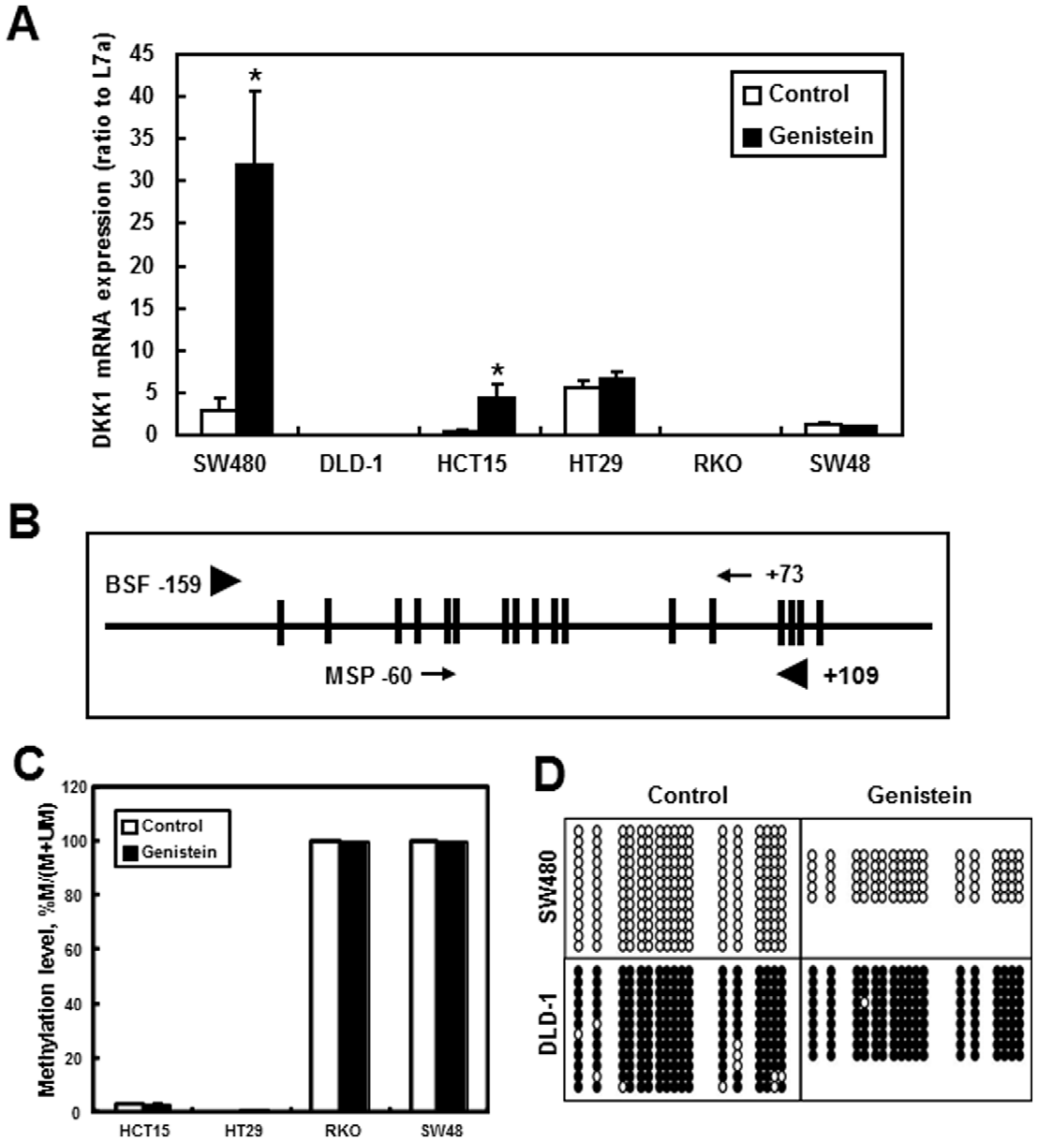
DNA methylation of Comparison of DKK1 expression promoter in colon cancer cell line SW480, DLD-1, HCT15, HT29, RKO and SW48. SW480, DLD-1, HCT15, HT29, RKO and SW48 cells were treated with genistein for 2 d before sample collection for further analysis. A) Relative DKK1 mRNA expression. mRNA expression was analyzed using RT-PCR. Data were normalized to internal control L7a. Three independent cell samples were analyzed and presented as the mean ± SEM. Asterisks (*) indicate statistical significance compared to control within the same cell line (p<0.05). B) Schematic drawing of the DKK1 promoter region. Horizontal bar represents the CpG island within the promoter region. The vertical bars indicate individual CpG sites. Black arrowheads indicate the positions of the primers used for in PCR amplifications for Bisulfite Sequencing (−159 to +109). Arrows represent the positions of the primers used for PCR amplifications in MSP (−60∼+73). C) MSP of the DKK1 promoter region in HCT15, HT29, RKO and SW48. Y-axis represents the methylation level and the values are calculated as percentage of M/(M+UM). When not seen, error bars are within the columns. D) Bisulfite sequencing of the DKK1 promoter region in SW480 and DLD-1 cells. Each open circle represents an unmethylated C within a CpG while a filled circle represents a methylated C within a CpG. Each row of circles represents an individual clone used for sequencing.

To investigate the correlation between DNA methylation at the *DKK1* promoter CpG island and the effect of genistein treatment on *DKK1* expression, real-time RT-PCR was performed to measure *DKK1* mRNA expression and methylation specific polymerase chain reaction (MSP) and bisulfite sequencing (BSF) of the *DKK1* promoter region were performed to examine the promoter methylation status (Fig. 1). First, *DKK1* gene expression was analyzed in available colon cancer cell lines to investigate the effects from genistein treatment. Among the cell lines tested, SW480 and HCT15 cells showed induction of DKK1 gene expression by genistein (Fig. 1A). Analysis of the DNA methylation at the *DKK1* promoter region confirmed that DKK1 gene expression in general is inversely related to the methylation level of the region tested (−159/+109, Fig. 1B, 1C, and 1D). Specifically, RKO, SW48, and DLD-1 cells exhibited very high, if not complete, methylation of the region (Fig. 1C, and 1D). This hypermethylation is inversely related to the level of DKK1 gene expression as shown in Fig. 1A. For the DKK1-expressing cell lines HCT15, HT29, and SW480, there is minimal DNA methylation observed (Fig. 1C and 1D). More importantly, genistein treatment did not alter the DNA methylation of the *DKK1* promoter region in any cell lines tested, regardless of the level of DNA methylation in the region (Fig. 1C and 1D).

### Genistein Dose- and Time-dependently Induced DKK1 Gene Expression

**Figure 2:**
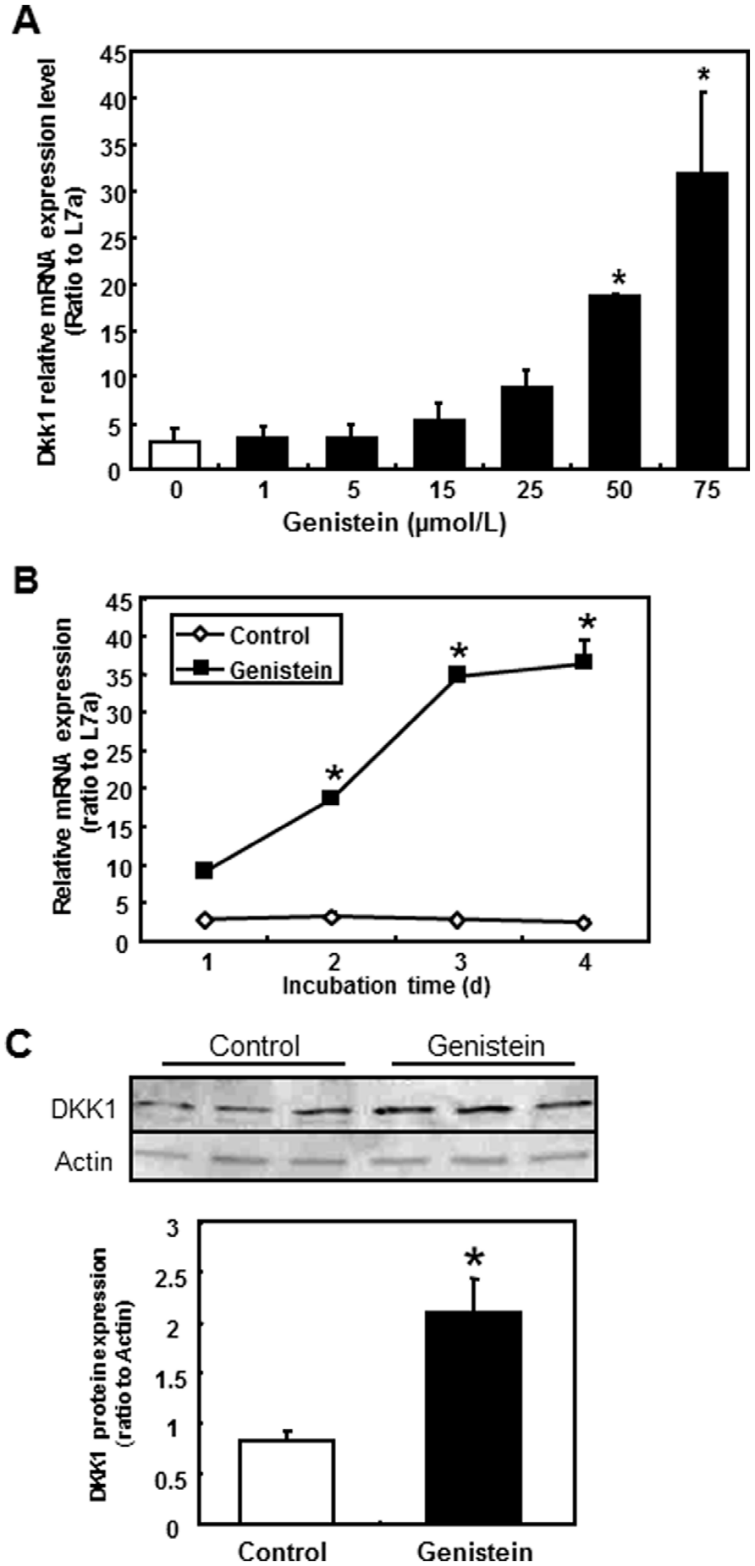
*DKK1* expression in colon cancer cell line SW480 following genistein treatments. **A**) SW480 cells were treated by various concentrations of genistein for 2 d. mRNA expression was analyzed by RT-PCR. X-axis indicates genistein concentrations and y-axis shows relative mRNA expression level. Data were normalized to internal control L7a. Asterisks (*) indicate statistical significance compared to 0 µmol/L genistein (*p*<0.05). **B**) Time course of *DKK1* expression in SW480 following genistein treatment. SW480 cells were treated with 75 µmol/L of genistein and sampled at day 1, 2, 3 and 4 of the treatment. Data were normalized to internal control L7a. Three independent experiments were performed and presented as the mean±SEM. Where invisible, the error bars are contained within the bars. Asterisks (*) indicate statistical significance compared to d 1 of 75 µmol/L genistein (*p*<0.05). **C)** DKK1 protein expression. Whole cell protein extracts were collected from SW480 cells and western blot analysis of DKK1 was performed as described in materials and methods. A blot from western analysis is shown and the quantification represents the mean± SEM from 3 independent samples. Actin was used as the loading control. Asterisks (*) indicate statistical significance compared to control 0 µmol/L genistein (*p*<0.05).

Real-time RT-PCR was performed to measure *DKK1* mRNA expression levels in response to different genistein doses and exposure times. Overall, *DKK1* expression was increased by genistein in a dose-dependent manner (Fig. 2A). After 2 d of genistein treatment, *DKK1* mRNA expression was 3-fold and 8-fold higher following the 50 and 75 µmol/L genistein treatments respectively, when compared to the no-genistein control (p<0.05, Fig. 2A). The mRNA level of *DKK1* in SW480 cells was also increased by genistein treatment (75 µmol/L) in a time-dependent manner (Fig. 2B) and reached a >10-fold induction at d 4 compared to the level in the DMSO control.

The induction of *DKK1* was confirmed by measuring its protein expression level in SW480 cells (Fig. 2C). DKK1 protein expression showed a 3-fold induction by 75 µmol/L of genistein treatment in SW480 cells following a 4-d treatment when compared to the control.

### Genistein Inhibited Cell Cycle Progression in SW480 Cells

**Figure 3:**
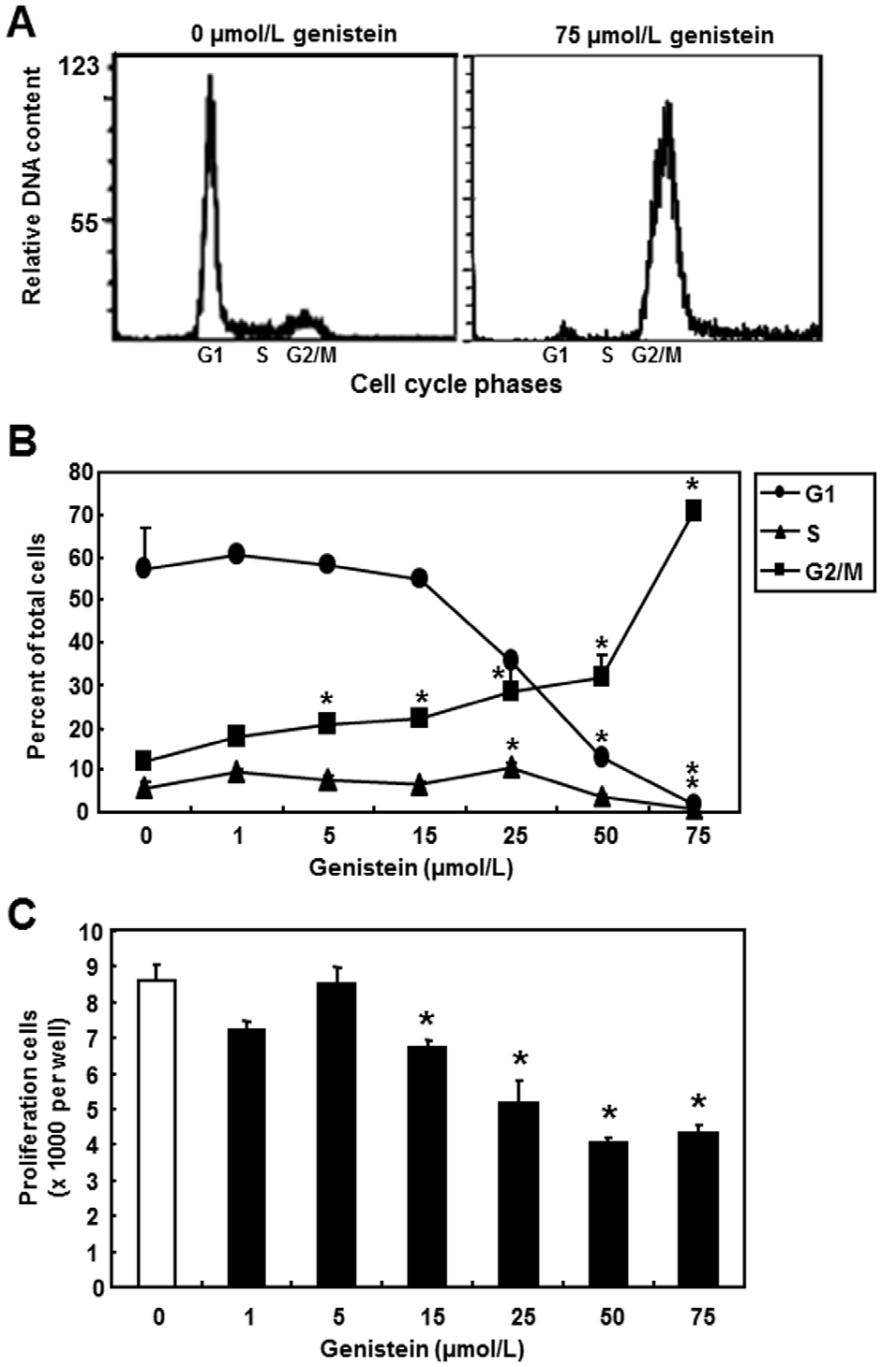
Effects of genistein treatment on cell physiology in SW480. SW480 cells were treated with various concentrations of genistein for 2 d before sample collection. **A**) Representative DNA histograms of genistein treatment of 0 and 75 µmol/L by flow cytometry. Y-axis represents the relative DNA content and x-axis shows cell cycle stages. **B**) Cell cycle analysis of genistein treated cells. The percentage of cells in G1 (•), S (▴) or G2/M (▪) is shown. Data represent the means ± SEM of three independent experiments. **C)** WST-1 proliferation assay. WST-1 signals from each well were read at 450 nm for absorbance against a reference wavelength of 630 nm. Absorbance was converted to actual cell numbers using a standard generated by serial dilutions of a known number of cells. Three independent cell samples were analyzed and presented as the mean ± SEM. Asterisks (*) indicate statistical significance compared to 0 µmol/L of genistein (*p*<0.05).

To investigate the effect of genistein on cell cycle progression of SW480 cells, the DNA content of SW480 cells was measured by area fit analysis after the flow cytometry analysis. Histograms showed that the population of cells at G1 was markedly decreased by 75 µmol/L of genistein treatment compared to the no-genistein control, with a decrease of cells in the S phase (Fig. 3A). On the other hand, genistein treatment resulted in an accumulation of cells at G2/M phase in SW480 cells.

The effects of genistein on cell cycle progression were further analyzed by treatment of cells with genistein in concentrations from 1 to 75 µmol/L. Percentage of cells in different phases was calculated as the ratio of gated cells to the total cell population. Genistein concentrations above 5 µmol/L increased the percentage of cells at G2/M phase significantly (p<0.05) with the highest accumulation observed at 75 µmol/L of genistein (p<0.05, Fig. 3B). The results demonstrate that the percentage of cells in the G1 phase was abolished following 75 µmol/L of genistein treatment (Fig. 3B). The percentage of S phase cells was significantly reduced (p<0.05) following 50 and 75 µmol/L genistein treatments after a transient increase by 25 µmol/L genistein when compared to the no-genistein control. Taken together, cell cycle progression in SW480 cells was regulated by genistein in a dose-dependent manner.

### Genistein Inhibited Cell Proliferation in SW480 and Several Other Colon Cancer Cell Lines

Cell proliferation was tested by the WST-1 assay, which showed that genistein treatments decreased the cell numbers of SW480 cells in a dose-dependent manner (Fig. 3C). At genistein concentrations above 15 µmol/L cell numbers were significantly decreased when compared to the no-genistein control (p<0.05). This anti-proliferative effect of genistein was confirmed in several colon cancer cell lines (Figure S1). The AnnexinV-propidium iodide assay showed no change by any of the genistein treatments, indicating that genistein does not affect the apoptosis rate (data not shown). These results indicate that growth-suppressing effect of genistein is generalizable to the common human colon cancer cell lines.

### Cyclin D1 mRNA Expression Decreased following Genistein Treatment in SW480 Cells

**Figure 4:**
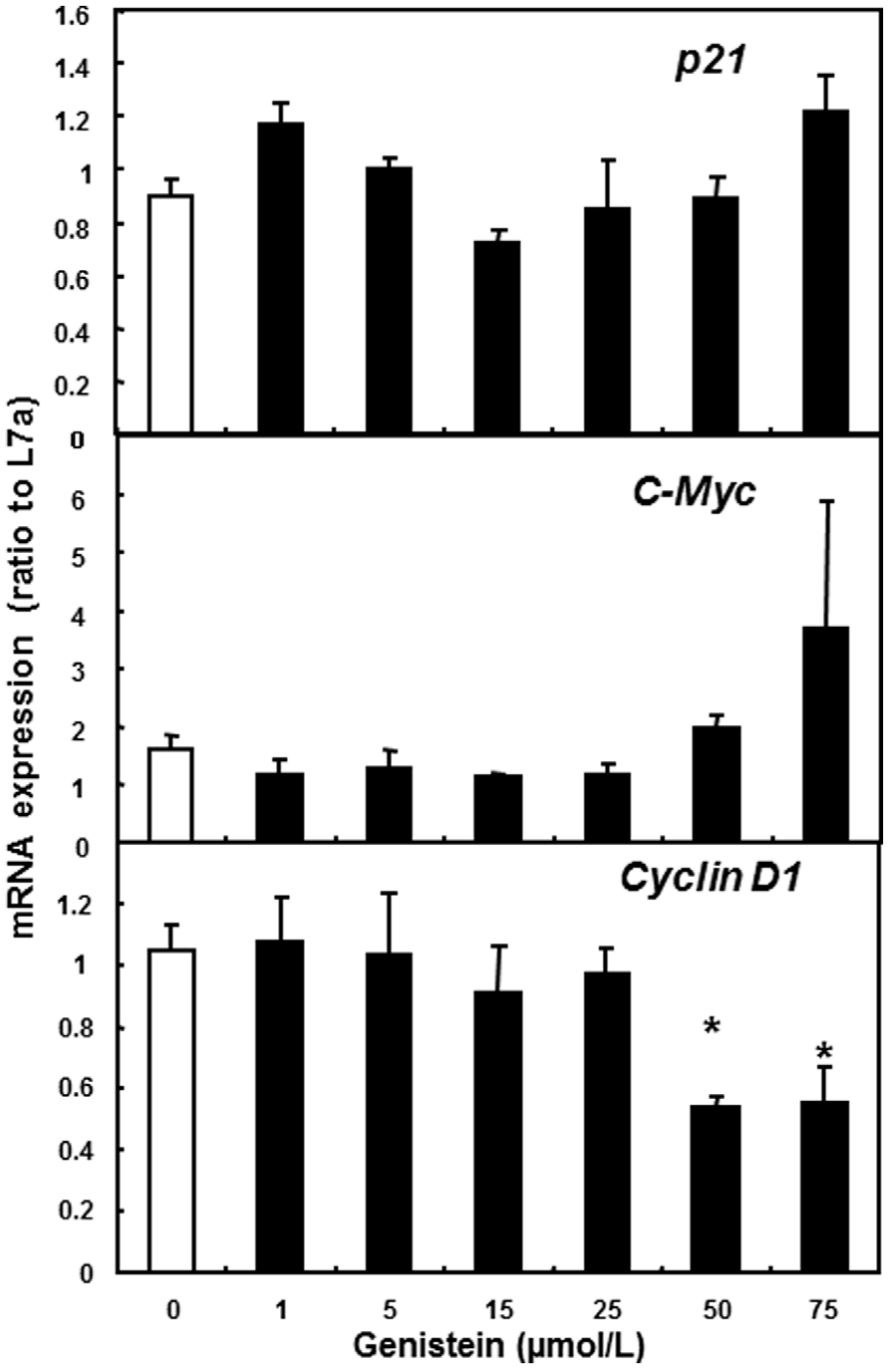
mRNA expressions of *p21*, *Cyclin D1* and *c-MYC* by genistein treatments. SW480 cells were treated with 0, 1, 5, 15, 25, 50 or 75 µmol/L of genistein for 2 d. The mRNA expression levels of *p21, Cyclin D1* and *c-MYC* were measured by RT-PCR. L7a was used as the internal control. Three independent experiments was analyzed and presented as the mean±SEM. Asterisks (*) indicate statistical significance compared to Control, 0 µmol/L genistein (*p*<0.05).

Cell cycle control genes *p21*, *Cyclin D1* and *c-MYC* are highly correlated to the regulation of cell growth. Based on the data presented above, cell cycle arrest occurred in G2 in SW480 cells by genistein treatment. To further investigate the effects of genistein on the cell cycle progression, the mRNA expression of cell cycle control genes *p21*, *Cyclin D1* and *c-MYC* were measured by real-time RT-PCR. Our results showed that the expression of *Cyclin D1* was decreased significantly (p<0.05) by 50 and 75 µmol/L of genistein compared to control (Fig. 4, p<0.05). The expression of *p21* and *c-MYC* was not affected by any of the genistein treatments. The decreased expression of *Cyclin D1* is in agreement with the cell cycle data from flow cytometry showing that cell cycle progression was inhibited by genistein treatment.

### Overexpression of DKK1 Inhibited Cell Cycle Progression and Cell Proliferation in SW480 Cells

**Figure 5:**
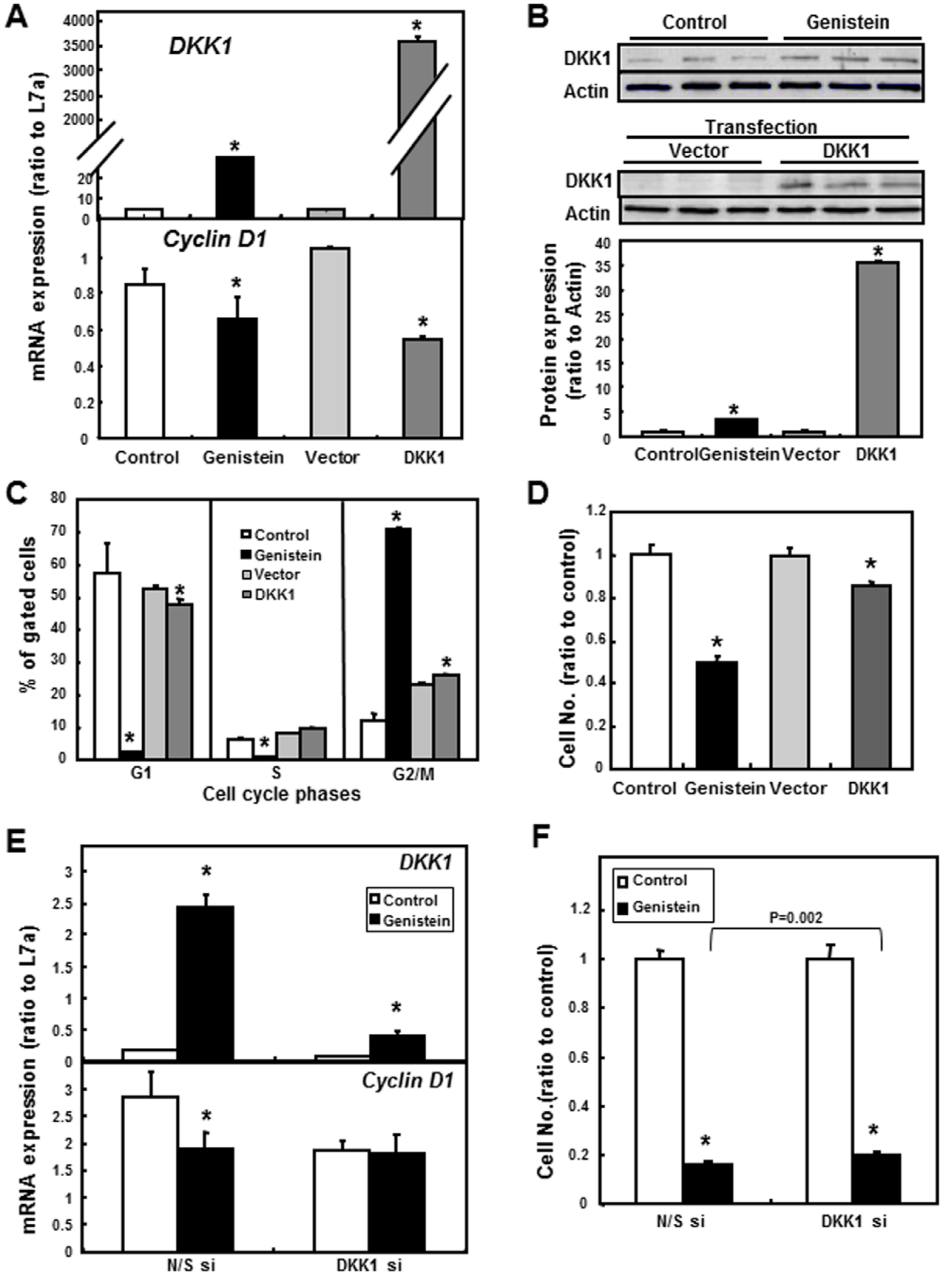
Overexpression and silencing of *DKK1* in SW480 cells. pCMV vector containing human *DKK1* gene was used to transfect cells before sample collection for analysis. Empty pCMV vector was used as the control for the overexpression experiments. Three independent cell samples were analyzed and presented as the mean ± SEM. **A**) mRNA expression of *DKK1* and *Cyclin D1* in regular and *DKK1*-transfected SW480 cells. The mRNA expression was analyzed by RT-PCR. Data were normalized to internal control L7a. **B**) DKK1 protein expression in regular cells and transfected SW480 cells. Whole cell protein extracts were collected from transfected SW480 cells and western blot analysis of DKK1 was performed as described in materials and methods. A representative blot is shown and the quantification represents the mean± SEM from 3 independent dishes. Actin was used as the loading control. **C**) Cell cycle analysis of regular and *DKK1*-transfected SW480 cells. Result was obtained by flow cytometry. Y-axis represents % of gated cells and x-axis shows different cell cycle stages, including G1, S and G2/M. **D**) WST-1 proliferation assay in vector- and *DKK1*-transfected SW480 cells. WST-1 signals were converted to actual cell numbers using a standard generated by serial dilutions of a known number of cells. Data were normalized to control for genistein treatment and vector for DKK1 transfection, respectively. Asterisks (*) indicate statistical significance compared with the respective control group (genistein to control; DKK1 to vector; *p*<0.05). For the knockdown of DKK1, siRNA duplexes against human DKK1 gene were transfected into SW480 cells. Scrambled sequences were used as the control siRNA. **E**) mRNA expression of *DKK1* and *Cyclin D1* in control siRNA (N/S si) and *DKK1* siRNA (DKK1 si)-treated SW480 cells by control and genistein treatments. The mRNA expression was analyzed by RT-PCR. Data were normalized to internal control L7a. **F**) WST-1 proliferation assay in control siRNA and *DKK1* siRNA SW480 cells. WST-1 signals were converted to actual cell numbers using a standard generated by serial dilutions of a known number of cells. Asterisks (*) indicate statistical significance compared to Control 0 µmol/L genistein (*p*<0.05). The bracket indicates statistical difference between the N/S si and DKK1 si groups treated with genistein (p = 0.002).

To investigate the potential role of *DKK1* in cell cycle progression, SW480 cells were transfected with a pCMV-XL5 plasmid containing human *DKK1*. Overexpression of *DKK1* was confirmed by RT-PCR analysis of mRNA and western blot analysis of protein (Fig. 5A and 5B). Analysis of *Cyclin D1* expression showed that overexpression of DKK1 induced similar repression of the gene expression as the genistein treatment (Fig. 5A). Overexpression of *DKK1* significantly increased the percentage of cells in the G2/M phase when compared to the vector control (p<0.05, Fig. 5C), although to a less extent compared to the genistein treatment. Meanwhile, the percentage of cells in the G1 phase was significantly decreased in cells overexpressing DKK1 (p<0.05), and there was no significant change in the number of cells in the S phase. Results from the WST-1 assay showed that overexpression of *DKK1* decreased the proliferation of SW480 cells, although to a much less extent compared to the genistein treatment (p<0.05, Fig. 5D). Overall, the overexpression of *DKK1* produced similar effects on inhibiting cell progression and cell proliferation as did the genistein treatment, suggesting a role of DKK1 in these cellular events.

### Knockdown of DKK1 in SW480 Cells Partially Reversed the Changes of Cellular Profile Caused by Genistein Treatment

To investigate whether the alteration of cellular properties of SW480 caused by genistein is dependent on the *DKK1* gene expression, siRNA targeting DKK1 was transfected into SW480 cells followed by genistein treatment. Real-time RT-PCR showed that siRNA effectively reduced DKK1 expression in SW480 in both control and genistein treatments (p<0.05, Fig. 5E). Moreover, the reduction of *Cyclin D1* mRNA expression by genistein treatment as observed in non-specific siRNA-treated cells (N/S si) was abolished by the knockdown of DKK1 (DKK1 si, Fig. 5E). Meanwhile, although the growth-inhibitory effect from genistein is observed in both N/S siRNA and *DKK1* siRNA groups, knockdown of DKK1 significantly increased the proliferation of SW480 cells compared to that of control siRNA in genistein treatment (p<0.05, DKK1 siRNA vs N/S siRNA in genistein treatments, Fig. 5F).

### Genistein Induced Histone Acetylation within the DKK1 Gene in SW480 and HCT15 Cells

**Figure 6:**
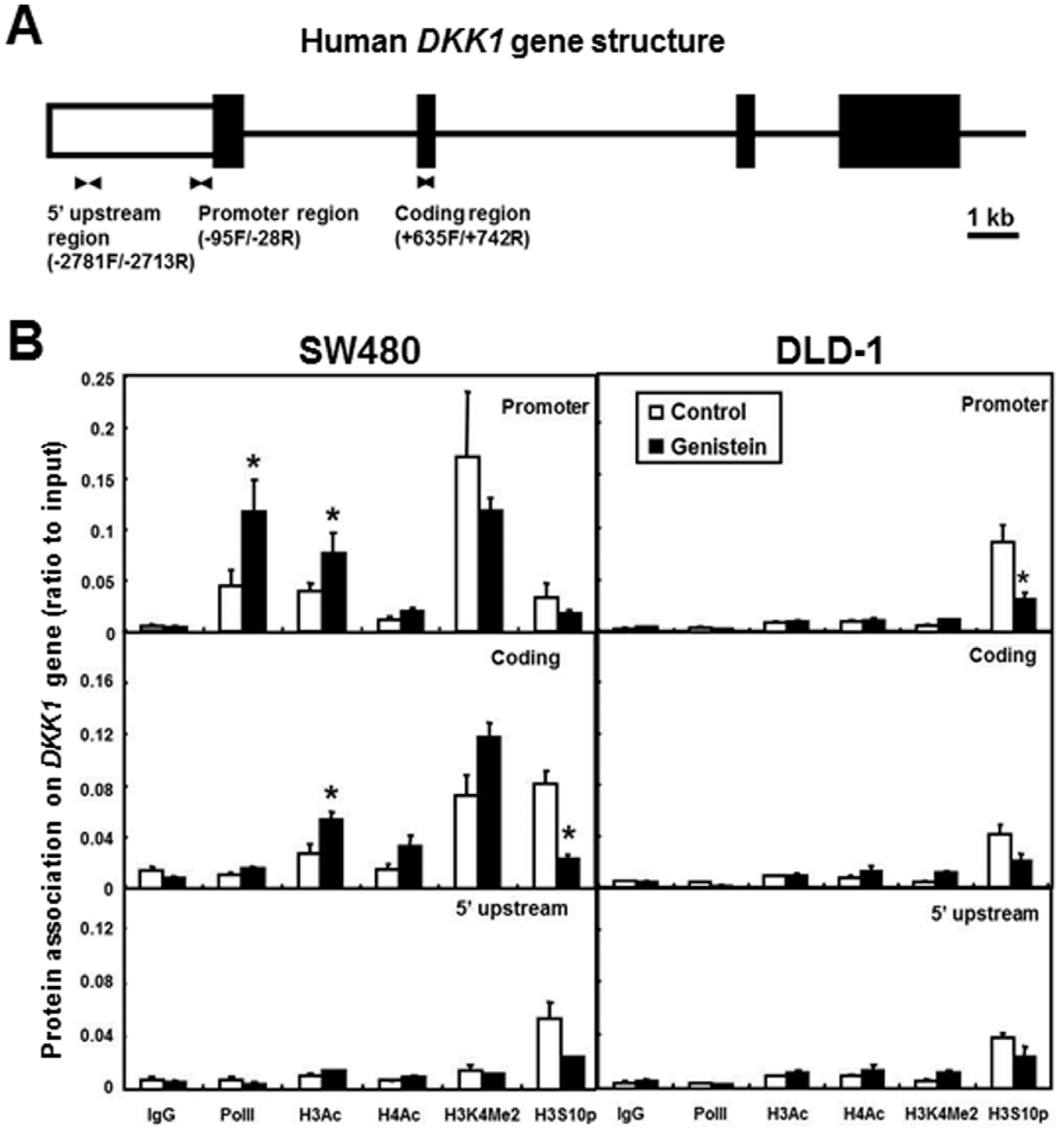
ChIP analysis of chromatin modifications. **A**) A schematic drawing of the *DKK1* gene. Black arrowheads represent primers used for PCR to test three regions in the ChIP assay: promoter, coding and 5′ upstream control. Filled boxes represent exons of the *DKK1* gene, while the open box represents the *DKK1* promoter. Black lines represent introns. **B**) ChIP analysis of the relative protein abundance within different regions of the *DKK1* gene in SW480 and DLD-1 cells. Immunoprecipitated DNA was analyzed by real time PCR. Specific antibodies used for immunoprecipitation are labeled on the x-axis. A nonspecific rabbit IgG was used as the negative control. Data were plotted as the ratio to the value from 25% of input DNA. Three independent experiments were analyzed and presented as the mean ± SEM. Asterisks (*) indicate statistical significance compared with the Control using the same antibody within the cell line (p<0.05).

ChIP was performed to analyze the chromatin structure at the promoter (-96/−28), coding (+635/+742), and 5′ upstream control regions (−2781/−2713) of the *DKK1* gene (Fig. 6A). The 5′ upstream control region was used as a negative control to show the relative inactive region during transcription. DLD-1 cells were used as a negative control to show the chromatin status when the *DKK1* gene expression is silenced and not responsive to genistein treatment. Normal rabbit IgG was used as an internal control antibody and any DNA binding similar to the binding in the control IgG was considered to be non-specific. Increased RNA polymerase II (PolII) binding at the *DKK1* promoter region indicated increased transcription of the *DKK1* gene in SW480 cells in response to genistein (p<0.05, Fig. 6B). In contrast, there was minimal PolII binding within the *DKK1* gene in DLD-1 cells, confirming the silenced transcription of the gene in DLD-1 cells (Fig. 6B).

To determine whether the increased transcription level of the *DKK1* gene in response to genistein was modulated by alterations of chromatin structure, antibodies against acetylated, methylated, or phosphorylated histones were used in the ChIP assay. Histone modifications at the three representative regions of the *DKK1* gene were first examined in SW480 cells after the genistein treatment, using DLD-1 cells as the negative control. Increases in acetylated histone H3 at the promoter and coding regions of *DKK1* were observed in SW480 cells after genistein treatment (p<0.05, Fig. 6B, H3Ac), with no significant acetylation of histones in DLD-1 cells before or after genistein treatment (Fig. 6B). The abundance of the other acetylated histone tested, the acetylated histone H4, was not affected by genistein treatment in either cell lines (H4Ac). Although there was a much higher level of dimethyl histone H3 lysine 4 in SW480 cells compared to DLD-1 cells, genistein treatment did not affect the methylation status of this histone H3 residue (Fig. 6B, H3K4Me2). Phosphorylation of histone H3 at serine 10 was also investigated and the result showed that there was a modest decrease at various regions of the *DKK1* gene by genistein treatment in all cell lines tested (p<0.05, Fig. 6B, H3S10p). In summary, the active transcription of the *DKK1* gene was associated with the increased histone H3 acetylation at its gene region, which likely resulted in the recruitment of PolII to its promoter.

### DKK1 mRNA Expression was Induced by Histone Deacetylase (HDAC) Inhibitor in SW480 Cells

**Figure 7:**
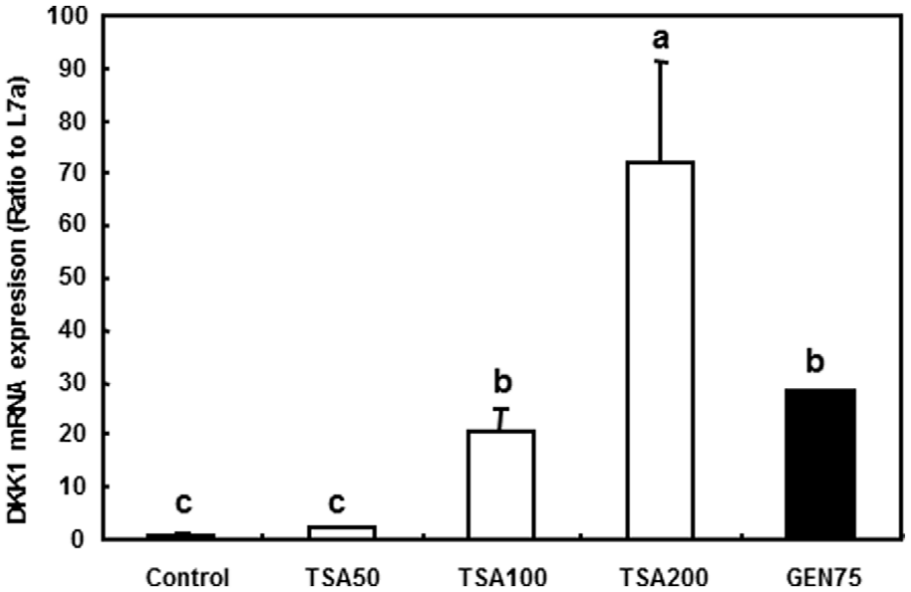
*DKK1* mRNA expression after TSA treatment. *DKK1* mRNA level was analyzed in SW480 cells treated with 50, 100 or 200 nmol/L of TSA (TSA50, TSA100, and TSA200, respectively) for 1 d. Genistein treatment was done as previously described. mRNA expression level was analyzed using RT-PCR. Three independent cell samples were analyzed and presented as the mean±SEM. Values with different letters differ (*p*<0.05).

TSA is a histone deacetylase (HDAC) inhibitor that interacts with most of the HDAC family members and induces acetylation of histones. To confirm the effects of genistein-induced histone acetylation on *DKK1* transcription, we treated SW480 cells with a series of TSA concentrations and compared the results to genistein treatment. The mRNA expression of *DKK1* was increased significantly by the TSA treatment in a dose-dependent manner, similar to the result observed from genistein treatment (p<0.05, Fig. 7).

## Discussion

The present study intended to identify the potential mechanisms of the antitumor effects of genistein. We observed that both mRNA and protein levels of DKK1 were upregulated by genistein treatment. Our results demonstrated that genistein treatment induced cell cycle arrest and inhibited cell proliferation in SW480 colon cancer cells. This was later confirmed in several other colon cancer cell lines. Both overexpression and knockdown of *DKK1* confirmed the involvement of DKK1 in the inhibition of cell cycle progression and cell proliferation. Our result demonstrated that increased DKK1 expression might be one of the reasons that caused the cell cycle arrest and inhibition of cell proliferation by genistein treatment. Furthermore, comparison between the *DKK1*-expressing cells including HCT15, HT29 and SW480, and the *DKK1*-repressed cells including RKO, SW48 and DLD-1 showed that gene transcription of *DKK1* is inversely related to its promoter methylation status. Genistein does not affect DNA methylation of the *DKK1* promoter. On the other hand and more importantly, we have made the observation that genistein induces histone acetylation within the *DKK1* gene and this is correlated with the activation of its gene transcription.

The anticancer roles of genistein, including promotion of apoptosis, inhibition of cell cycle progression, cell proliferation and invasion, the blockage of angiogenesis and metastasis in various types of cancers have been well documented [7], [36], [37], [38]. The molecular mechanisms behind these anticancer functions of genistein include modulations of cell cycle inhibitors, the regulation of apoptosis-related genes, as well as the inhibition of IGF-IR and PI3K/AKT signaling [11], [39], [40], [41]. Work by our group has shown that genistein inhibited another critical pathway in colon cancer development, the WNT/β-catenin pathway, by interfering with the promoter methylation of specific genes. Specifically, genistein induced *WNT5a* expression by decreasing methylation within the gene’s promoter region [27]. Genistein also inhibited the WNT/β-catenin pathway by activating another WNT antagonist, *sFRP2*, by demethylating CpG islands within the gene [26]. The current study expanded the knowledge to include DKK1 as another factor in WNT signaling that mediates the response from genistein treatment. As a repressor of cancer cell growth, DKK1 is a main antagonist that interferes with the WNT pathway and downregulates the expression of the downstream target genes including Cyclin D1 [28], [42], [43].

The expression of DKK1 was silenced by promoter hypermethylation in the advanced stage of colon cancer (Dukes’ C and D) [34]. In DLD-1 cell line from Dukes’ C, a more advanced stage of colon cancer, demethylation of the DKK1 gene by 5-aza-2′-deoxycytidine, a DNMT inhibitor, re-activated DKK1 expression [34], [44]. Since genistein has been shown to activate genes through CpG demethylation [19], we first tested the possibility of DKK1 promoter demethylation by genistein. Our result confirmed that the methylation level at the promoter CpG island is inversely related to the gene expression of DKK1. In the cell lines with hypermethylated promoter region, including DLD-1, RKO, and SW48, there was minimal if not silenced DKK1 gene expression. Furthermore, genistein treatment did not change the hypermethylation of the region in these cells, nor did it induce any gene expression. On the other hand, DKK1 was unmethylated in the early stage of colon cancer (Dukes’ B) and related cell lines including SW480 [34] and HCT15. Both MSP and bisulfite sequencing confirmed that the DNA methylation was very little, if any, in the cell lines. Furthermore, the hypomethylation is not changed by genistein treatment. Therefore, DNA demethylation was ruled out as a mechanism for the induction of DKK1 gene expression by genistein in these cell lines.

It has been reported that genistein also modulates other epigenetic markers at chromatin level [16], [45], [46]. Histone acetylation results in a more open chromatin structure, which allows for transcription factor binding and enhanced transcriptional activity of the target genes. Histone deacetylation has been shown to contribute to the silencing of genes [47], [48]. In our study, we observed the enhanced binding of RNA polymerase II and acetylation of histone H3 at the *DKK1* promoter, in accordance with the activation of *DKK1* transcription by genistein. We showed that little histone acetylation occurred within the *DKK1* gene in DLD-1 cells, indicating a less active and repressed promoter, in both control and genistein treated cells. HDAC inhibitor treatment also confirmed the association between upregulation of DKK1 and induction of histone acetylation by genistein. This is in agreement with the previous discovery on DKK1 regulation by histone acetylation [47], [48] and further provides a potential mechanism of regulation of *DKK1* gene expression by genistein.

The dimethylation of histone H3 lysine 4 occurs ubiquitously in active euchromatins but not in heterochromatins [49]. When compared to DLD-1 cells that had very little dimethylation of histone H3 lysine 4, we observed a high level of dimethylation of histone H3 lysine 4 within the promoter and coding regions of *DKK1* in SW480 cells. Therefore this raises an important point regarding dimethyl histone H3 lysine 4 as a marker for a more dynamic chromatin structure rather than a marker for euchromatin in general. On the other hand, genistein had little effect on the dimethylation of histone H3 lysine 4 in either cell line. Interestingly a report by Santos-Rosa et al showed a better predictive value using trimethyl histone H3 lysine 4 for active gene expression [49]. Therefore, further study is required to examine the effects of genistein on other potential histone markers.

Phosphorylation of histone H3 is another modification associated with increased transcriptional activity of proto-oncogenes, and might therefore enhance tumorigenesis [50]. Our result showed a general decrease in phosphorylated H3 serine 10 by genistein within the *DKK1* gene in the colon cancer cell lines tested. While this is consistent with the anticancer activity of genistein, the explicit role of histone phosphorylation, especially at different regions of the *DKK1* gene, remains to be further evaluated.

In conclusion, we not only showed that in *DKK1*-expressing colon cancer cells, genistein-mediated histone acetylation contributed to the enhanced transcriptional activity of *DKK1*, but also demonstrated that genistein did not affect the DNA methylation pattern of the *DKK1* promoter regardless cancer developmental stage. The present study will contribute to the identification of the mechanistic basis behind the anticancer effects of genistein by presenting a novel relationship between the activation of *DKK1* expression and histone acetylation at the gene region in early stage of colon cancer development.

## Materials and Methods

### Cell Culture and Treatments

The human colon cancer cell lines SW480, DLD-1, HCT15, HT29, RKO and SW48 were purchased from ATCC (Manassas, VA). Minimum essential medium (MEM) was manufactured by the Cell Media Facility at the University of Illinois. Unless otherwise mentioned, all general chemicals and laboratory supplies were obtained from Fisher Scientific (www.fishersci.com). Cell culture ware was purchased from Sarstedt (Newton, NC). SW480 and DLD-1 cells were cultured in MEM, supplemented with 10% fetal bovine serum (FBS) and 1% antibiotic-antimycotic solution (ABAM) at 37°C in a humid incubator with 5% CO_2_. All experiments were performed using cells with 2–6 passages.

For testing the effects of genistein dosage, SW480 cells were treated in triplicate with genistein-containing media of 0, 1, 5, 15, 25, 50 or 75 µmol/L for 2 to 4 d, and cell samples were collected for mRNA expression, cell cycle and cell proliferation analysis. For western blot, DNA methylation, and ChIP analysis, cells were treated with 75 µmol/L of genistein (GEN75).

### Quantitative Real-time PCR

Following genistein treatment, cells were harvested in TriReagent (Sigma-Aldrich, St Louis, MO) according to the manufacturer’s instructions. mRNA concentration was measured by SmartSpec Plus spectrophotometer (BioRad laboratories Inc, Irvine, CA) at 260 nm. Total mRNA (2 µg) was used for cDNA synthesis using High Capacity cDNA Reverse transcription Kit (Applied Biosystems, Foster City, CA) in a DNA 2720 Thermal Cycler (Applied Biosystems, Foster City, CA). The program was as follows: 25°C for 10 min, 37°C for 120 min, and 85°C for 5 s. After the reaction, 25 ng of samples were used for quantitative real-time PCR and gene expression levels were determined using 2x Perfecta SYBR Green fast master mix (Quanta BioSciences, www.vwr.com) in a 7300 real-time PCR system (Applied Biosystems, Foster City, CA). Primer sequences for the experiment were designed using Vector NTI software (Invitrogen Corporation, Carlsbad, CA) and primers were synthesized by Integrated DNA Technologies (www.idtdna.com). The sequences of the primers used are as follows: *L7a*, forward, 5′-TTTGGCATTGGACAGGACATCC-3′ and reverse, 5′-AGCGGGGCCATTTCACAAAG-3′; *DKK1*, forward, 5′-GATCATAGCACCTTGGATGGG-3′ and reverse, 5′-GGCACAGTCTGATGACCGG-3′; *p21*, forward, 5′-CCGCGACTGTGATGCGCTAATG-3′ and reverse, 5′-CTCGGTGACAAAGTCGAAGTC-3′; *c-MYC*, forward, 5′-GCTCCTGGCAAAAGGTCAGAGTCT-3′ and reverse, 5′-ACCAGTGGGCTGTGAGGAGGTT-3′ and *Cyclin D1*, forward, 5′-CGCCCTCGGTGTCCTACTTCAA-3′ and reverse, 5′-GTGGCGACGATCTTCCGCAT-3′. The reaction was as follows: 95°C for 15 min to activate Taq polymerase followed by 40 cycles of 95°C for 15 s and 60°C for 60 s. After amplification, dissociation curves were acquired by stepwise increases from 55°C to 95°C to ensure a specific product was amplified in the reaction. Standard curves with slope of −3.30±0.20 and R^2^≥0.99 were accepted. Human *L7a* was used as an internal control to normalize the raw data.

### Western Blotting Analysis

After treatments, cells were lysed in 500 µL lysis buffer (0.125 mol/L Tris–HCl, pH 6.8, 1% SDS, 0.04% bromophenol blue, and 20% glycerol, v/v ) with 1x proteinase inhibitor (Roche applied sciences, Indianapolis, IN) and phosphatase inhibitor cocktail 1 and 2 (Sigma-Aldrich, St. Lois, MO). Total protein was size-fractionated on a 12% Tris–HCl polyacrylamide gel and transferred onto a PVDF membrane (BioRad laboratories Inc, Irvine, CA) at 0.3 A. The PVDF membrane was incubated with blocking solution containing 10% (w/v) nonfat dry milk (NFDM), 20 mmol/L Tris–HCl, pH 7.6, 137 mmol/L NaCl, and 0.1% (v/v) Tween-20 for 1 h at room temperature. A rabbit polyclonal antibody against DKK1 (sc-25516, Santa Cruz, CA) was diluted to 1∶1,000 in the blocking solution with 10% NFDM and incubated with the membrane for 3 h at room temperature. Subsequently, the membrane was washed with blocking solution containing 5% NFDM for 5×5 min. A goat anti-rabbit HRP-conjugated secondary antibody was diluted to 1∶10,000 in the blocking solution containing 5% NFDM and incubated with the membrane for 1 h at room temperature. After washing for 5×5 min in the blocking solution containing 1% (w/v) NFDM, the membrane was exposed to the enhanced chemiluminescence reagent Super Signal West Dura (Thermo Fisher Scientific, Rockford, IL). Signals from the membrane were detected and quantified by ChemiDoc XRS imaging system (Bio-Rad, Hercules, CA). Actin (sc-1616-R, Santa Cruz, CA) was used as a loading control.

### Cell Cycle Analysis by Flow Cytometry

At the end of the 2-d genistein treatment as described above, cells were trypsinized, centrifuged at 1000×g, and re-suspended in 0.5 mL 1×PBS. For fixation, 0.5 mL 100% ice-cold ethanol was added to each sample and incubated for a minimum of 20 min. After centrifugation at 1000×g for 6 min, ethanol was decanted and 0.5 mL 1x PBS containing 50 µg/mL Propidium Iodide (PI) and 100 µg/mL RNase A was added to the pellet and mixed. Samples were incubated at room temperature in the dark for a minimum of 20 min before analysis by BD LSR II flow cytometer (BD Bioscience, Bedford, MA). Data were analyzed using FCS 3.0 software.

### Cell Proliferation Analysis Using WST-1 Assay

At the end of the 2-d genistein treatment as described above, 10 µL cell proliferation reagent, WST-1 (Roche applied science, Indianapolis, IN) was added to each well of the 96-well plate and incubated for 4 h. At the same time, a serial dilution of cells were mixed with 10 µL WST-1 reagents and aliquoted to the unused wells of the same plate as the standards for converting the absorbance readings to cell numbers. The plate was shaken thoroughly for 1 min. The absorbance of samples was read at 450 nm against a reference wavelength of 630 nm in a microplate reader (ELX800, BioTek, Winooski, VT).

### DKK1 Overexpression

SW480 cells were cultured as described above and transfected with either the control pCMV vector or *DKK1*-containing pCMV (OriGene, Rockville, MD). The empty pCMV vector was used as a transfection control. After transfection with Superfect Transfection Reagent (Qiagen, Valencia, CA), cells were replenished with fresh MEM and incubated for 48 h before sample collection.

### DKK1 Knockdown

SW480 cells were plated at 0.5 ×10^6^ per well in 6-well plate or 3000 per well in 96-well plates and incubated overnight at 37°C with 5% CO_2_. At the end of 2-d genistein treatment as described above, transfection was performed. At the time of transfection, 300 nmol/L of siRNA against human scramble sequence (N/S si, QIAGEN, Valencia, CA) or 300 nmol/L of siRNA against human DKK1 (*DKK1* si, Integrated DNA Technologies) was prepared in 200 µL serum-free media (SFM). The siRNA solution was incubated with 7 µL DharmaFect#2 (in 200 µL SFM, Dharmacon, www.fishersci.com) at room temperature for 20 min to form the transfection complex. Each well of the 6-well plates and the 96-well plates was replenished with 1.6 mL and 80 µL antibiotic-free MEM including DMSO or genistein (final concentration of genistein is 75 µmol/L), respectively before the transfection complex was added (400 µL/well for 6-well plates and 20 µL/well for 96-well plates). The transfected cells were incubated at 37°C for 2-d for subsequent mRNA, protein, cell cycle and cell proliferation analysis. siRNA duplex for DKK1 used is a combination of three duplexes as follows: 5'- rGrCrCrGrGrArUrArCrArGrArArArGrArUrCrArCrCrArUrCrA- 3', and 5'-rUrGrArUrGrGrUrGrArUrCrUrUrUrCrUrGrUrArUrCrCrGrGrCrArA- 3' (HSC.RNAI.N012242.12.1); 5'- rGrUrArUrCrArCrArCrCrArArArGrGrArCrArArGrArArGrGrT- 3', and 5'- rArCrCrUrUrCrUrUrGrUrCrCrUrUrUrGrGrUrGrUrGrArUrArCrArU- 3' (HSC.RNAI.N012242.12.2); 5'- rArGrArArCrGrGrArArGrUrGrUrGrArUrArUrGrUrUrUrArArA- 3', and 5'- rUrUrUrArArArCrArUrArUrCrArCrArCrUrUrCrCrGrUrUrCrUrUrG- 3' (HSC.RNAI.N012242.12.3, Integrated DNA Technologies).

### Methylation Specific PCR (MSP)

HCT15, HT29, RKO and SW48 cells were plated and treated as mentioned above. On d 2 of genistein treatment, cells were scraped and collected by centrifugation. Genomic DNA was isolated from the cell lines using GenElute genomic DNA Kit (Sigma-Aldrich, St. Louis, MO) and DNA concentration was measured by 260 nm absorbance. Total genomic DNA (2 µg) was treated with sodium bisulfite reagent using the EZ Methylation-Gold kit (Zymo Research, Orange, CA). In each MSP reaction, 10 ng of final product was used in a 20 µL volume containing 10 µL 2x Perfecta SYBR Green fast master mix (Quanta BioSciences, www.vwr.com) and 0.25 µmol/L of each primer in a 7300 real-time PCR system (Applied Biosystems, Foster City, CA). For human DKK1, one pair of methylated (M) primers (MSP DKK1 M −60∼+73) and one pair of unmethylated (U) primers covering the same region (MSP DKK1 U −62∼+73) were used to analyze DNA methylation at the 5′ proximal region within the CpG island predicted by CpGPlot (www.ebi.ac.uk/emboss/cpgplot/). Methylation level is calculated as the percentage of M/(M+U). The primers used in MSP are: MSP-M, forward, 5′- TTAAGGGGTCGGAATGTTTC -3′, reverse, 5′- TCGACTACAAAATCAAAATCCG -3′, and MSP-U, forward, 5′-TTTTTAAGGGGTTGGAATGTTTT -3′, reverse, 5′- TTCAACTACAAAATCAAAATCCAAA -3′.

### Bisulfite Sequencing

Genomic DNA was isolated from SW480 and DLD-1 cells using DNeasy Tissue Kit (Qiagen, Valencia, CA). Bisulfite conversion was performed with 1.4 µg of total genomic DNA by EZ Methylation-Gold kit (Zymo Research, Orange, CA) according to the manufacturer’s instructions. After purification, 200 ng of converted DNA was used as a template for each PCR reaction. Promoter CpG island for *DKK1* was predicted as being located at -223 to +122 (http://www.ebi.ac.uk/emboss/cpgplot/) [34] and primers were designed to amplify the -159 to +109 region (forward, 5′-GTGAAGAGTGTTAAAGGTTTTTTTT-3′ and reverse, 5′-CTTTACAAACCTAAATCCCCAC-3′). The amplicons from the PCR were cloned using TOPO-PCR cloning kit (Invitrogen, Carlsbard, CA) and sequenced at the Biotechnology Center at the University of Illinois (Urbana, IL). The C in a CpG is unmethylated when sequencing result shows a “T” and methylated when sequencing result shows a “C”.

### Chromatin Immunoprecipitation (ChIP)

ChIP analysis was performed according to a modified protocol [51]. SW480 and DLD-1 cells were treated with 75 µmol/L genistein solution for 4 d before harvest. Cross-linking was performed using 1% formaldehyde for 10 min at room temperature. Nuclei were collected after nuclei swelling buffer treatment (5 mmol/L PIPES pH 8.0, 85 mmol/L KCl, 0.5% NP40) and lysed in SDS lysis buffer (50 mmol/L Tris-HCl pH 8.1, 10 mmol/L EDTA, 1% SDS) containing protease and phosphatase inhibitor cocktails. The chromatin was sonicated using a Sonic Dismembrator (model F100, Fisher Scientific, Pittsburgh, PA) with power set at 5 on ice for 5 bursts of 40 s with 2 min cooling between each burst. The average length of sonicated chromatin was measured by agarose gel electrophoresis to be ∼500 bp. Cell debris was removed by centrifugation at 13,000×g for 10 min at 4°C. Sheared chromatin was diluted to 10 mL with ChIP dilution buffer. One mL of diluted chromatin was incubated overnight at 4°C with 2 µg of each antibody. The chromatin-antibody complex was precipitated with 60 µL of 50% slurry of pre-blocked protein G-agarose beads (Millipore, Billerica, MA) for 2 h. A normal rabbit IgG was used as a negative control. Supernatant from the normal rabbit IgG was saved as the input DNA for each sample. The protein G-agarose beads were washed sequentially with 1 mL each of the following solutions: low salt (20 mmol/L pH 8.0 Tris-HCl, 0.1% SDS, 2 mmol/L EDTA, 150 mmol/L NaCl, 1% triton X-100), high salt (20 mmol/L pH 8.0 Tris-HCl, 0.1% SDS, 2 mmol/L EDTA, 500 mmol/L NaCl, 1% triton X-100), LiCl (10 mmol/L pH 8.0 Tris-HCl, 0.25 mmol/L LiCl, 1% NP40, 1 mmol/L EDTA, 1% sodium deoxycholate) and twice TE (pH 8.0). Antibody/protein/DNA complexes were eluted by 2×250 µL elution buffer (1% SDS and 50 mmol/L NaHCO_3_) at 37°C for 15 min each. Reverse cross-linking was performed with 20 µL of 5 mol/L NaCl and 1 µg of RNase A at 65°C for 5 h. Chromatin DNA was purified using QiaPrep miniprep kit (Qiagen, Valencia, CA) after proteinase K digestion. Immunoprecipitated DNA was detected by real time RT-PCR using primers targeting different regions of the DKK1 gene: promoter region: forward, 5′-GGCTTTGTTGTCTCCCTCCCAAG-3′ and reverse, 5′-CCACCGCGGCTGCCTTTATA-3′; coding region, forward, 5′-GATCATAGCACCTTGGATGGG-3′ and reverse, 5′-GGCACAGTCTGATGACCGG-3′ and 5′ upstream control region, forward, 5′-ACTGGCTGCTGATGGAACAATGAG-3′ and reverse, 5′-TCATTGGCAACAAGAGTGCAAGGT-3′. The antibodies used are as follows: from Millipore, acetylated histone H3 (H3Ac, 06-599), acetylated histone H4 (H4Ac, 06-866), dimethyl-histone H3 lysine 4 (H3K4me2, 07-030); from Santa Cruz Biotechnologies, normal rabbit IgG (IgG, sc-2027), RNA polymerase II (PolII, sc-899) and phosphorylated histone H3 serine 10 (H3S10p, sc-8656R).

### Trichostatin A (TSA) Treatment

SW480 cells were plated at 0.2 ×10^6^ per dish in a 60 mm culture dish in regular MEM containing 10% FBS and 1% ABAM. After overnight culture, cells were treated with culture media containing 50, 100 or 200 nmol/L TSA for 1 or 2 d. Total mRNA was isolated and mRNA expression was analyzed by real time RT-PCR as described above.

### Statistical Analysis

Unpaired two-tailed Student’s *t* test was performed for mRNA and protein expression, cell proliferation and cell cycle analyses, and Methylation Specific PCR. Two-way ANOVA using LSMeans was performed for data from ChIP (SAS Institute Inc., Cary, NC). One-way ANOVA and Tukey test was applied to compare the significance in levels among different groups in Fig. 4F and Fig. 7. Data are presented as means ± SEM. Statistical significance was set as p<0.05.

## Supporting Information

Figure S1 **Cell proliferation in colon cancer cells.** WST-1 proliferation assay was performed in DLD-1, HCT15, HT29, RKO and SW48 cells. WST-1 signals were converted to actual cell numbers using a standard generated by serial dilutions of a known number of cells. Data were normalized to respective control for genistein treatment. Y-axis represents relative cell number (% to control). Asterisks (*) indicate statistical significance compared with the control group of the same cell line (p<0.05).
(TIFF)
