# Supplementary figures and images for: Genistein Affects Histone Modifications on Dickkopf-Related Protein 1 (DKK1) Gene in SW480 Human Colon Cancer Cell Line
Source: PLoS One. 2012 Jul 18;7(7):e40955. doi: 10.1371/journal.pone.0040955 (PMC3399800; doi:10.1371/journal.pone.0040955)

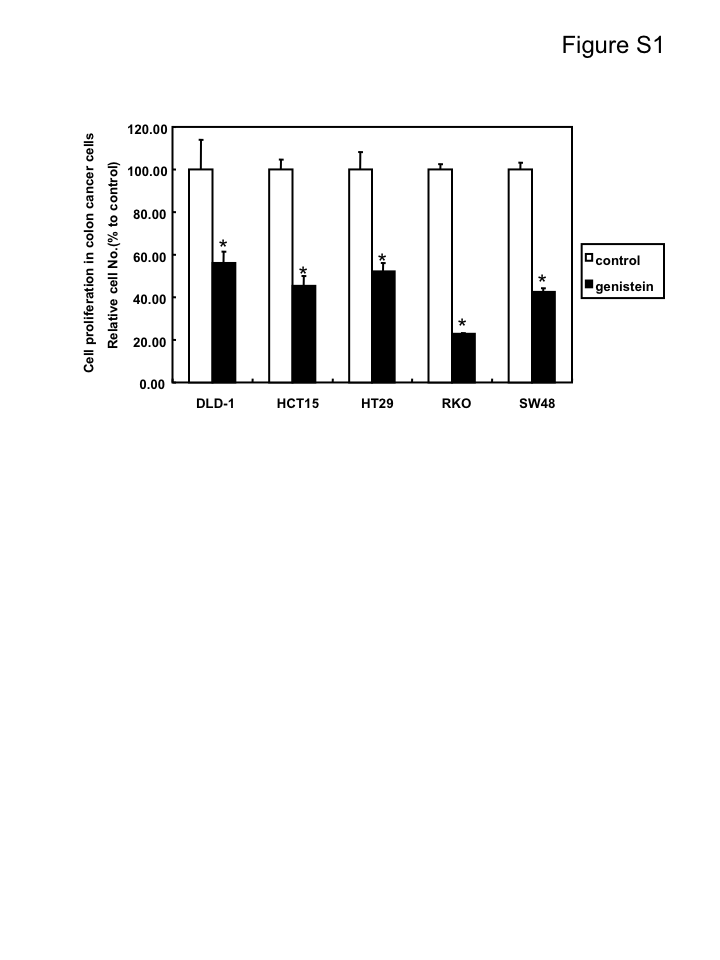

Supplement: Figure S1 — Cell proliferation in colon cancer cells. WST-1 proliferation assay was performed in DLD-1, HCT15, HT29, RKO and SW48 cells. WST-1 signals were converted to actual cell numbers using a standard generated by serial dilutions of a known number of cells. Data were normalized to respective control for genistein treatment. Y-axis represents relative cell number (% to control). Asterisks (*) indicate statistical significance compared with the control group of the same cell line (p<0.05). (TIFF) [file pone.0040955.s001.tiff]
